# Complete Genome Sequence of a Pantón-Valentine Leukocidin-Negative Community-Associated Methicillin-Resistant *Staphylococcus aureus* Strain of Sequence type 72 from Korea

**DOI:** 10.1371/journal.pone.0072803

**Published:** 2013-08-20

**Authors:** Yan Chen, Som S. Chatterjee, Stephen F. Porcella, Yun-Song Yu, Michael Otto

**Affiliations:** 1 Pathogen Molecular Genetics Section, Laboratory of Human Bacterial Pathogenesis, National Institute of Allergy and Infectious Diseases, U.S. National Institutes of Health, Bethesda, Maryland, United States of America; 2 Department of Infectious Diseases, Sir Run Run Shaw Hospital, College of Medicine, Zhejiang University, Hangzhou, China; 3 Genomics Unit, Research Technologies Branch, National Institute of Allergy and Infectious Diseases, U.S. National Institutes of Health, Hamilton, Montana, United States of America; Huashan Hospital, Shanghai Medical College, Fudan University, China

## Abstract

In the past decade, community-associated (CA-) infections with methicillin-resistant *Staphylococcus aureus* (MRSA) have emerged throughout the world. Different CA-MRSA strains dominate in different geographical locations. Many CA-MRSA lineages contain genes coding for the Pantón-Valentine leukocidin. However, the role of this leukotoxin in CA-MRSA pathogenesis is still controversial. The genome sequences of two key PVL-positive CA-MRSA strains (USA300, USA400) have been reported, but we lack information on the more recently found PVL-negative CA-MRSA strains. One such strain is the PVL-negative ST72, the main cause of CA-MRSA infections in Korea. Here, we report the entire genome sequence of CA-MRSA ST72 and analyze its gene content with a focus on virulence factors. Our results show that this strain does not have considerable differences in virulence factor content compared to other CA-MRSA strains (USA300, USA400), indicating that other toxins do not substitute for the lack of PVL in ST72. This finding is in accordance with the notion that differential expression of widespread virulence determinants, rather than the acquisition of additional virulence factors on mobile genetic elements, such as PVL, is responsible for the increased virulence of CA- compared to hospital-associated MRSA.

## Introduction

*Staphylococcus aureus* is a dangerous human pathogen and *S. aureus* infections are among the most frequent causes of deaths in hospitals around the globe [1]. Antibiotic resistance severely complicates the treatment of such infections [2]. After the worldwide spread of penicillin-resistant strains in the mid of the last century, methicillin became the treatment option of choice for *S. aureus* infections. However, methicillin resistance developed quickly, and nowadays methicillin-resistant *S. aureus* (MRSA) is pandemic, with many countries reporting methicillin resistance rates among hospital-associated *S. aureus* isolates that exceed 50% [3].

In the 1990s, MRSA infections – previously limited to predisposed patients in hospitals – started occurring in otherwise healthy people in the community without connections to the hospital setting [4]. These community-associated (CA-) MRSA infections are on a worldwide surge, with the United States so far seeing the most pronounced CA-MRSA epidemic. Most CA-MRSA infections are moderately severe infections of the skin and soft tissues, but more severe and sometimes fatal infections, such as necrotizing pneumonia, are also seen with CA-MRSA. The rise of CA-MRSA is due to the development of strains that combine methicillin resistance with a high level of aggressive virulence not commonly present in hospital-associated (HA-) MRSA [5]. Globally, CA-MRSA infections are caused by different lineages that are not genetically related [6]. In addition to pronounced virulence, which they all share, some strains may express specific additional factors that further promote pathogenic success. For example, the epidemic U.S. strain, USA300, harbors a mobile genetic element (MGE), called arginine catabolic element (ACME), containing a gene, *speG*, which abrogates the unique hypersensitivity of *S. aureus* to host-produced polyamines, thereby increasing survival on the human skin and during skin abscesses [7].

Soon after the first cases of CA-MRSA infections, researchers started determining the genetic composition of CA-MRSA isolates, including by whole-genome sequencing [8,9]. The most important initial finding was that genes encoding a specific leukotoxin, the Pantón-Valentine leukocidin (PVL), were present in virtually all CA-MRSA isolates, while these genes, called *lukS* and *lukF*, are much less frequent among HA-MRSA [10]. However, further investigation using animal infection models indicated that the extraordinary virulence of CA-MRSA is only in part, and in comparatively rare types of infections such as severe lung infection, due to PVL [5,11,12]. Rather, high expression of core genome-encoded virulence determinants, such as phenol-soluble modulins (PSMs) and α-toxin, appears to have played a preeminent role in the evolution of CA-MRSA virulence, especially as it relates to skin infections [12–14].

In addition to animal experiments casting doubt on the key role of PVL and the acquisition of the prophage ΦSLT containing the *lukSF* genes during the evolution of CA-MRSA virulence, several CA-MRSA strains have been isolated in the meantime that do not harbor *lukSF* and therefore do not produce PVL [5]. One such strain is the CA-MRSA clone of sequence type (ST) 72 that is the premier cause of CA-MRSA infections in Korea [15]. Here, to gain insight in the genetic composition as a basis for the extraordinary, PVL-independent virulence of that strain, we determined the whole genome sequence of a CA-MRSA ST72 isolate and analyzed its composition with a focus on virulence factors.

## Materials and Methods

### DNA extraction

Total bacterial DNA was isolated from an overnight culture of HL1 (ST72) using lysostaphin digestion and the method of Marmur [16]. The pellet of a 1-ml overnight culture was resuspended with 400 µl buffer P1 (Qiagen), to which 20 µl lysostaphin were added. The sample was incubated at 37°C for 15 min. Then 20 µl of a saturated sodium dodecylsulfate solution (in 45% ethanol) was added, the sample was vortexed, and incubated at 37°C for 5 min. Then 130 µl of a 5 M sodium perchlorate solution were added, and the sample was vortexed again. Six hundred µl of phenol/chloroform/isoamylalcohol (25:24:1 by volume) were added, the sample was vortexed, and the bottom phase transferred to a new tube. This extraction was repeated twice, then 800 µl ice-cold ethanol was added to precipitate the DNA, which was harvested by centrifugation for 15 min at 13,000 rpm in a tabletop centrifuge. The DNA was washed twice with 70% ethanol and resuspended in water.

### Genome sequencing, annotation and comparative analysis

DNA sequencing was performed using fragment and paired-end libraries on a Roche 454 FLX genome sequencer using Titanium chemistry (454 Life Sciences [a Roche company], Branford, CT), Coverage of 40-60× or higher was obtained according to the manufacturer’s recommendations. Reads were assembled using the GS Assembler Version 2.5 software program. 454 reads were re-aligned to the contigs to check for assembly accuracy, and misassembled portions were corrected All gaps between contigs were closed by oligonucleotide primer design, PCR fragment generation, and Sanger sequencing of the PCR products on an Applied Biosystems 3730XL DNA sequencer (Applied Biosystems, Foster City, CA), Primer walking of large gaps or correction of ambiguous base calls was performed by PCR and Sanger sequencing. Open reading frame (ORF) calling was performed using public and proprietary algorithms, with a minimum length cutoff of 40 amino acids, as previously described [17,18]. The genome sequence and annotation of HL1 (ST72) are deposited in DDBJ/EMBL/GenBank under the NCBI accession numbers CP003979 (chromosome), CP003980 (pHL1), CP003981 (pHL2). ORFs displaying evidence of frameshifts or mutations leading to premature stop codons were identified by proprietary algorithms and were manually verified. Genome comparisons were performed using ClustalW alignments.

### Genome analysis

To compare genome contents, we used a proprietary algorithm (Integrated Genomics) and compared every ORF from the HL1 genome with the ORFs from the two selected *S. aureus* genomes (FPR3757, MW2). Each ORF was compared using three metrics: similarity score (1e-10), functional annotation, and protein length. For ORFs to be considered for inclusion the following criteria had to be satisfied. The considered ORFs must have a P-Score similarity to an ORF from the HL1 genome of 1e-10 or less. In addition, the ORF in consideration should either have same functional annotation as of the HL1 genome’s ORF or have at least 80% matching protein length with that of the corresponding ORF in the HL1 genome. Further genome analyses were performed using ERGO (Integrated Genomics).

## Results and Discussion

### Overview

The CA-MRSA isolate HL1, previously also termed CN1 [13], was obtained from the pus of a necrotizing fasciitis infection in an 80-year old patient in the Seoul area of South Korea [15]. The isolate was then determined to be PVL-negative, resistant to clindamycin and erythromycin, belong to ST72 and spa type t324, and harbor SCC*mec* type IVa. Furthermore, we previously showed that it has high virulence in a rabbit model of skin infection and promotes neutrophil lysis to an extent almost as pronounced as seen with strain USA300 and within the range of other global CA-MRSA isolates [13].

The isolate has a genome of 2,757,070 base pairs (bp), with 2,726 assigned ORFs, of which 1970 have assigned functions, 53 tRNAs, and 9 rRNAs. The overall GC content is 32.79%. The HL1 genome is thus a little shorter than those of the prominent CA-MRSA strains USA300 (strain FPR3757, 2,917,469 bp, 2672 ORFs) and USA400 (strain MW2, 2,820,462 bp, 2644 ORFs) [4,8], which will serve as comparison in this study. HL1 also harbors two plasmids, which we named pHL1 and pHL2, of 3332 and 2472 bp, respectively.

### Virulence factors and pathogenicity islands

HL1 harbors the vSa**α**, vSa**β**, and vSa**γ** genomic islands and the **Φ**Sa3 prophage (Tab. 1). The vSa**α**, vSa**β**, and vSa**γ** genomic islands occur in most *S. aureus* strains and harbor a series of virulence factors. For example, vSa**α** encodes a series of exotoxins and a restriction/modification system. vSa**β** encodes another restriction/modification system, four serine proteases, and the bicomponent leukocidin LukDE. vSa**γ** encodes exotoxins, fibrinogen-binding proteins, a formyl peptide receptor 1 inhibitory protein, **α**-toxin, and the phenol-soluble modulins (PSMs) PSM**β**1 and PSM**β**2. The **Φ**Sa3 prophage is not always present in *S. aureus*; for example, it is absent from the HA-MRSA strain COL, representing the archaic MRSA lineage. It contains genes encoding immune evasion factors, namely the chemotaxis inhibitory protein CHIPS, the complement inhibitor SCIN, and staphylokinase. Of note, **Φ**Sa3 splits the gene encoding the sphingomyelinase **β**-toxin into two non-functional parts.

**Table 1:** Genomic and pathogenicity islands.

	HL1 (ST72)	MW2 (USA400)	FPR3757 (USA300)
vSa1	-	-	-
vSa2	-	-	-
vSa3	-	+	+
vSa4	-	+	-
vSa**α**	+	+	+
vSaβ	+	+	+
vSaγ	+	+	+
ΦSa1	-	-	-
ΦSa2	-	+	+
ΦSa3	+	+	+

**Figure 1:**
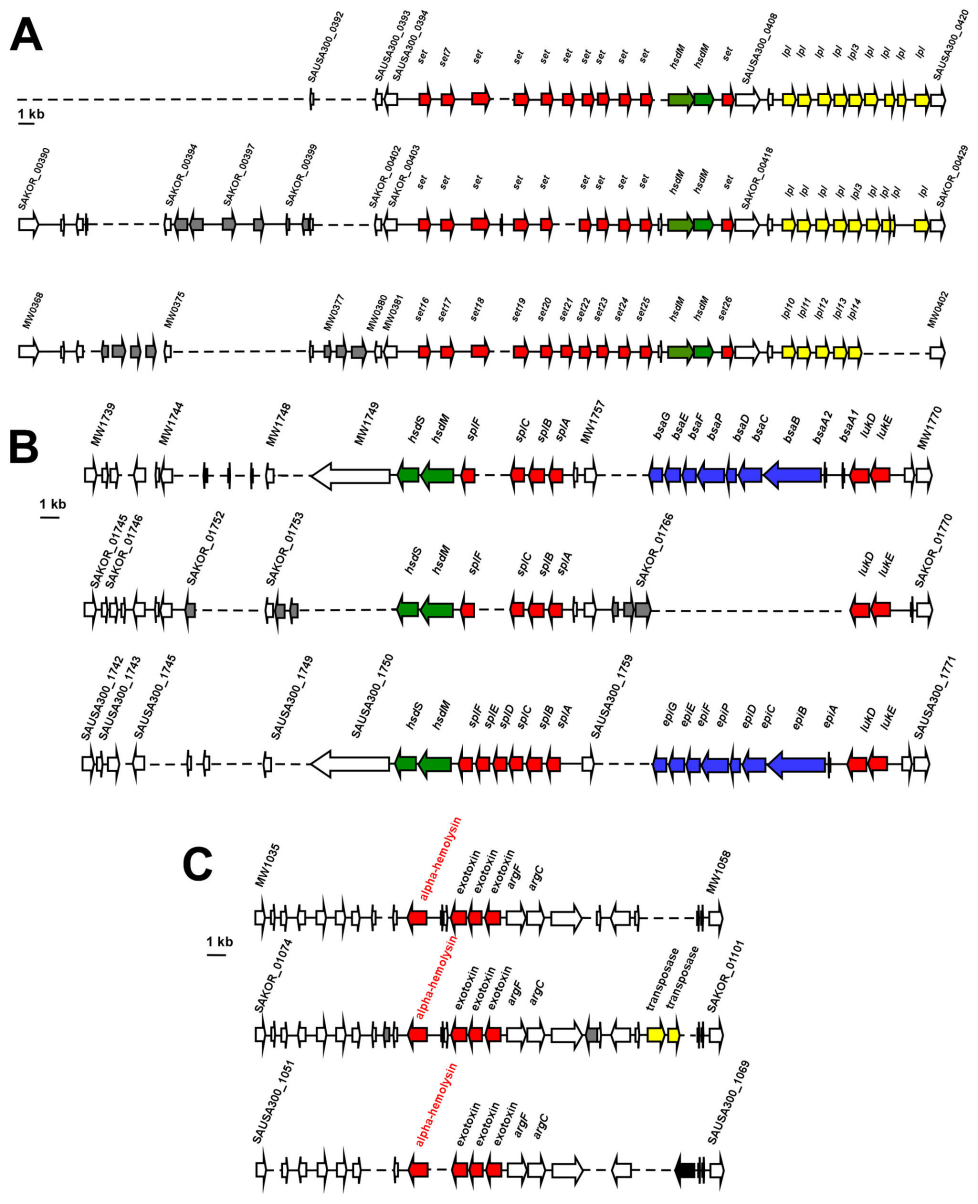
Genomic islands in ST72 HL1 compared to USA300 and USA400. A, vSaα genomic island. B, vSaβ genomic island. C, vSaγ genomic island. Broken lines, missing ORFs; red, virulence determinants; green, type I restriction-modification system specificity subunit; yellow, lipoproteins; blue, bacteriocin-related genes; yellow, determinants; grey= ORF not identical to other strains.

**Figure 2:**
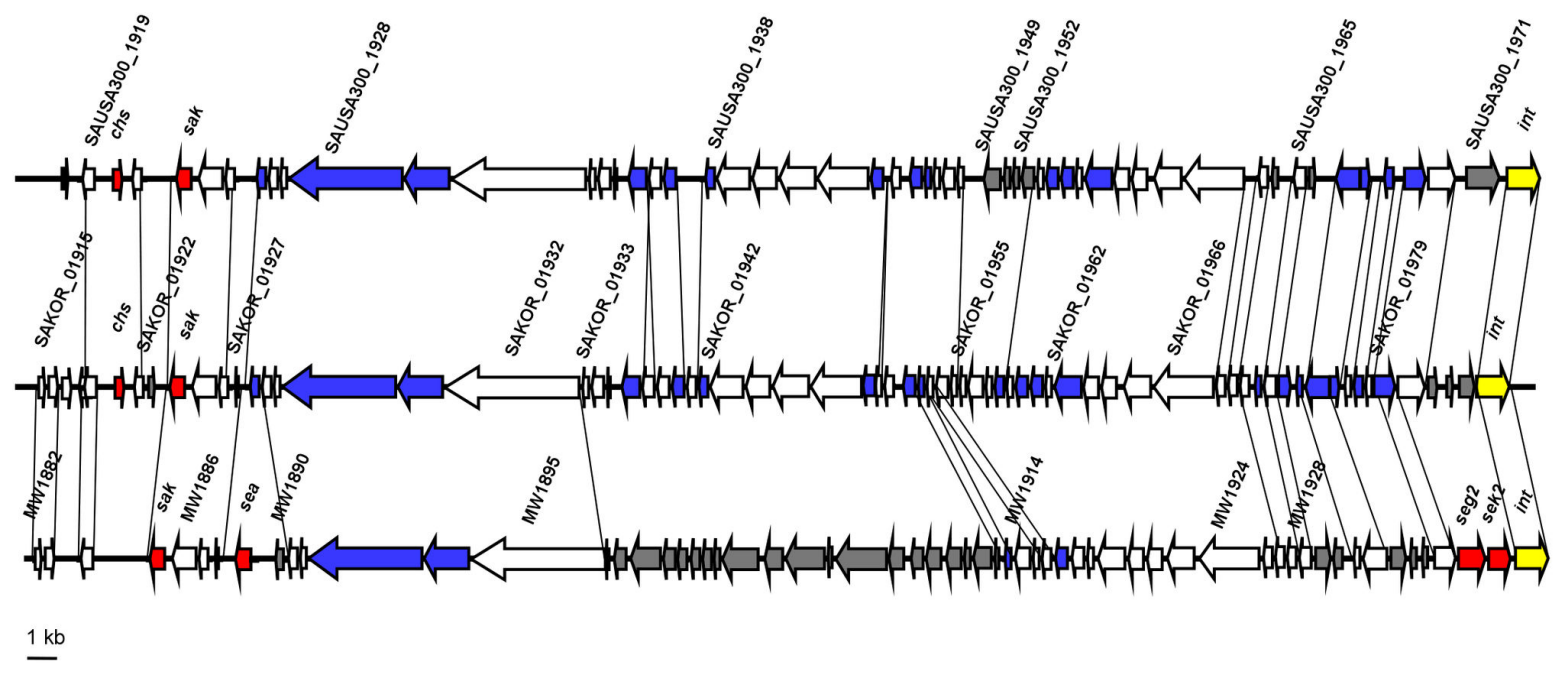
ΦSa3 prophage in ST72 HL1 compared to USA300 and USA400. Broken lines, missing ORFs; red, virulence determinants; blue, phage gene or phage-related gene; grey, ORF not identical to other strains.

These islands and the ΦSa3 prophage are also present in the USA300 and USA400 CA-MRSA strains. Although the overall genetic composition differs, there are no considerable differences in known virulence determinants (Figs. 1, 2). As a notable exception, HL1 vSaβ does not contain the *bsa* operon coding for the biosynthesis of the epidermin-like lantibiotic aureodermin. It has been shown that this operon encodes a functional lantibiotic [19]. However, production levels are very low under all conditions tested so far, and the role of aureodermin in *S. aureus* physiology is unclear [20]. Importantly, HL1 does not contain virulence factors encoded on genomic islands or prophages that are absent from USA300 and USA400. Vice versa, both USA300 and USA400 contain the ΦSa2 (ΦSLT) prophage harboring the *lukSF* genes coding for PVL. USA300 and USA400 also contain the vSa3, and USA400 the vSa4 pathogenicity islands, which are absent from HL1. vSa3 contains two enterotoxin genes with unknown function in virulence, and vSa4 does not comprise known virulence factors. Finally, USA300 contains ACME, harboring the recently described virulence and colonization factor *speG* [21].

A genome-wide analysis on 103 virulence genes to determine whether virulence factors present in HL1 are also present in USA300 and USA400 further confirmed that the overall composition of virulence genes in HL1, USA300, and USA400 is almost identical (Tab. 2). USA300 is missing two enterotoxin genes on vSaβ present in USA400 and HL1 (Fig. 1); and USA400 lacks the chemotaxis-inhibiting protein (CHIPs, gene *chs*), present in HL1 and USA300 on the ΦSa3 prophage (Fig. 2). The gene coding for the staphylococcal complement inhibitor SCIN is truncated in HL1, but not in USA300 or USA400. The gene *crtN* coding for the biosynthesis of the carotenoid staphyloxanthin immune evasion factor is annotated as truncated in HL1, because it appears to be split in a very short gene and a larger gene. However, the larger gene likely codes for the functional, previously described CrtN protein [22], while the shorter ORF may be a pseudo-gene. Similarly, HL1 and USA400 have a split coagulase gene, whereas it is not split in USA300. Finally, 28 surface proteins, identified by the sortase substrate LPXTG motif [23], did not show differences in composition between the three analyzed strains (Tab. 3).

**Table 2:** Presence/absence of important virulence factor genes found in HL1 in comparison to USA300 and USA400.

Annotation	Gene number	HL1	USA 400	USA300
Immunoglobulin G binding protein A precursor	SAKer_00085	+	+	+
Lipase (EC 3.1.1.3)	SAKer_00314	+	+	+
Exotoxin	SAKer_00368	+	+	+
Exotoxin	SAKer_00404	+	+	+
Exotoxin	SAKer_00405	+	+	+
Exotoxin	SAKer_00406	+	+	+
Exotoxin	SAKer_00408	+	+	+
Exotoxin	SAKer_00409	+	+	+
Exotoxin	SAKer_00410	+	+	+
Exotoxin	SAKer_00411	+	+	+
Exotoxin	SAKer_00412	+	+	+
Exotoxin	SAKer_00413	+	+	+
Exotoxin	SAKer_00417	+	+	+
Phenol-soluble modulin α4	SAKer_05000	+	+	+
Phenol-soluble modulin α3	SAKer_05001	+	+	+
Phenol-soluble modulin α2	SAKer_05002	+	+	+
Phenol-soluble modulin α1	SAKer_05003	+	+	+
Staphylococcal accessory regulator SarA	SAKer_00611	+	+	+
Lipase (EC 3.1.1.3)	SAKer_00649	+	+	+
Staphylococcal accessory regulator homolog (SarX)	SAKer_00663	+	+	+
Sensory transduction protein kinase SaeS (EC 2.7.13.3)	SAKer_00702	+	+	+
Two-component response regulator SaeR	SAKer_00703	+	+	+
Staphylocoagulase precursor	SAKer_00791	×^1^	×	+
Thermonuclease (EC 3.1.31.1)	SAKer_00795	+	+	+
Staphostatin B	SAKer_00967	+	+	+
Staphopain (EC 3.4.22.48)	SAKer_00968	+	+	+
Glutamyl endopeptidase precursor (EC 3.4.21.19)	SAKer_00969	+	+	+
Heme uptake protein IsdB	SAKer_01050	+	+	+
Heme uptake heme-iron binding protein IsdA	SAKer_01051	+	+	+
Heme uptake cell surface protein IsdC	SAKer_01052	+	+	+
Heme uptake ABC transporter, membrane component IsdD	SAKer_01053	+	+	+
Heme uptake ABC transporter, heme-binding protein IsdE	SAKer_01054	+	+	+
Heme uptake ABC transporter, permease protein IsdF	SAKer_01055	+	+	+
Sortase B family protein	SAKer_01056	+	+	+
Formyl peptide receptor family 1 inhibitory protein Flipr	SAKer_01077	+	+	+
Alpha-hemolysin	SAKer_01084	+	+	+
Exotoxin	SAKer_01087	+	+	+
Exotoxin	SAKer_01088	+	+	+
Exotoxin	SAKer_01089	+	+	+
Phenol-soluble modulin β1	SAKer_01099	+	+	+
Phenol-soluble modulin β2	SAKer_01100	+	+	+
Peptide deformylase (EC 3.5.1.88)	SAKer_01142	+	+	+
Thermonuclease (EC 3.1.31.1)	SAKer_01259	+	+	+
Lysyltransferase (EC 2.3.2.3) MprF	SAKer_01297	+	+	+
Two-component sensor kinase ArlS (EC 2.7.13.3)	SAKer_01351	+	+	+
Two-component response regulator ArlR	SAKer_01352	+	+	+
Superoxide dismutase (EC 1.15.1.1)	SAKer_01500	+	+	+
Enterotoxin	SAKer_01549	+	+	+
Enterotoxin	SAKer_01550	+	+	+
Heme uptake receptor for hemoglobin-haptoglobin complexes IsdH	SAKer_01672	+	+	+
Staphylococcal accessory regulator homolog (Rot)	SAKer_01705	+	+	+
Serine protease (EC 3.4.21. -) SplD	SAKer_01758	+	+	+
Serine protease (EC 3.4.21. -) SplC	SAKer_01759	+	+	+
Serine protease (EC 3.4.21. -) SplB	SAKer_01760	+	+	+
Serine protease (EC 3.4.21. -) SplA	SAKer_01761	+	+	+
Leukocidin F subunit (LukD)	SAKer_01767	+	+	+
Leukocidin S subunit (LukE)	SAKer_01768	+	+	+
Enterotoxin	SAKer_01776	+	+	-
Enterotoxin	SAKer_01777	+	+	+
Enterotoxin	SAKer_01778	+	+	-
Enterotoxin	SAKer_01779	+	+	+
Enterotoxin	SAKer_01780	+	+	+
Enterotoxin	SAKer_01781	+	+	+
Staphopain (EC 3.4.22.48)	SAKer_01871	+	+	+
Phenol-soluble modulin export ABC transporter permease PmtD	SAKer_01904	+	+	+
Phenol-soluble modulin export ABC transporter ATP-binding protein PmtC	SAKer_01905	+	+	+
Phenol-soluble modulin export ABC transporter permease PmtB	SAKer_01906	+	+	+
Phenol-soluble modulin export ABC transporter ATP-binding protein PmtA	SAKer_01907	+	+	+
Phospholipase C (beta-hemolysin) (EC 3.1.4.3), truncated	SAKer_01914	×	×	×
Staphylococcal complement inhibitor SCIN	SAKer_01918	×	+	+
Chemotaxis-inhibiting protein	SAKer_01920	+	-	+
Staphylokinase precursor	SAKer_01923	+	+	+
Leukocidin F subunit	SAKer_01986	+	+	+
Leukocidin S subunit	SAKer_01987	+	+	+
Delta-hemolysin	SAKer_01999	+	+	+
Accessory gene regulator protein B AgrB	SAKer_02000	+	+	+
Autoinducing peptide precursor AgrD	SAKer_02001	+	+	+
Sensory transduction histidine kinase AgrC (EC 2.7.3. -)	SAKer_02002	+	+	+
Accessory gene regulator protein A AgrA	SAKer_02003	+	+	+
RNA polymerase sigma-B factor	SAKer_02029	+	+	+
Staphylococcal accessory regulator homolog (SarV)	SAKer_02230	+	+	+
Staphylococcal accessory regulator homolog (SarR)	SAKer_02260	+	+	+
Staphylococcal accessory regulator homolog (SarY)	SAKer_02262	+	+	+
Esterase (EC 3.1.1. -)	SAKer_02324	+	+	+
Gamma-hemolysin subunit A	SAKer_02399	+	+	+
Gamma-hemolysin subunit C	SAKer_02401	+	+	+
Gamma-hemolysin subunit B	SAKer_02402	+	+	+
Staphylococcal accessory regulator homolog (SarT/SarH3)	SAKer_02485	+	+	+
Staphylococcal accessory regulator homolog (SarU/SarH2)	SAKer_02486	+	+	+
Sortase A	SAKer_02517	+	+	+
Esterase (EC 3.1.1. -)	SAKer_02525	+	+	+
Staphyloxanthin biosynthesis protein CrtN (dehydrosqualene dehydrogenase)	SAKer_02552	+	+	+
Staphyloxanthin biosynthesis protein CrtM (dehydrosqualene synthase)	SAKer_02554	+	+	+
Staphyloxanthin biosynthesis protein CrtQ	SAKer_02555	+	+	+
Staphyloxanthin biosynthesis protein CrtP	SAKer_02556	+	+	+
Staphyloxanthin biosynthesis protein CrtO	SAKer_02557	+	+	+
Esterase/Lipase (EC 3.1. -. -)	SAKer_02575	+	+	+
Zinc metalloproteinase aureolysin (EC 3.4.24.29)	SAKer_02638	+	+	+
N-acetylglucosaminyltransferase (EC 2.4.1. -) IcaA	SAKer_02668	+	+	+
N-acetylglucosaminyltransferase, accessory part IcaD	SAKer_02669	+	+	+
Polysaccharide intercellular adhesin deacetylase IcaB	SAKer_02670	+	+	+
Putative polysaccharide intercellular adhesin exporter IcaC	SAKer_02671	+	+	+
Lipase (EC 3.1.1.3)	SAKer_02673	+	+	+

1 truncated.

**Table 3:** LPXTG-motif surface proteins.

Annotation	Gene number	HL1	USA 400	USA 300
5'-nucleotidase (EC 3.1.3.5)	SAKer_00023	+	+	+
Immunoglobulin G binding protein A precursor	SAKer_00085	+	+	+
Hypothetical protein	SAKer_00110	+	+	+
Fibronectin-binding protein SdrC	SAKer_00549	+	+	+
Fibronectin-binding protein SdrD	SAKer_00550	+	+	+
Fibronectin-binding protein SdrE	SAKer_00551	+	+	+
Fibronectin-binding protein ClfA	SAKer_00790	+	+	+
Extracellular matrix binding protein / Fibrinogen-binding protein	SAKer_00793	+	+	+
Peptidoglycan endo-beta-N-acetylglucosaminidase (EC 3.2.1.96) / N-acetylmuramoyl-L-alanine amidase (EC 3.5.1.28)	SAKer_00974	+	+	+
Heme uptake protein IsdB	SAKer_01050	+	+	+
Heme uptake heme-iron binding protein IsdA	SAKer_01051	+	+	+
Heme uptake cell surface protein IsdC	SAKer_01052	+	+	+
Extracellular fibrinogen-binding protein	SAKer_01076	+	+	+
Extracellular fibrinogen-binding protein	SAKer_01079	+	+	+
Extracellular matrix binding protein	SAKer_01373	+	+	+
Elastin-binding protein EbpS	SAKer_01425	+	+	+
Heme uptake receptor for hemoglobin-haptoglobin complexes IsdH	SAKer_01672	+	+	+
Extracellular matrix binding protein	SAKer_01698	+	+	+
Outer membrane protein	SAKer_01913	+	+	+
Extracellular matrix binding protein	SAKer_02127	+	+	+
Para-nitrobenzyl esterase (EC 3.1.1. -)	SAKer_02435	+	+	+
Beta-N-acetylhexosaminidase (EC 3.2.1.52)	SAKer_02484	+	+	+
Fibronectin-binding protein FnbB	SAKer_02488	+	+	+
Fibronectin-binding protein FnbA	SAKer_02489	+	+	+
Clumping factor B	SAKer_02630	+	+	+
Hypothetical protein	SAKer_02647	+	+	+
Serine-rich adhesion for platelets SraP	SAKer_02648	+	+	+
Extracellular matrix binding protein	SAKer_02655	+	+	+

PSMs, short, amphipathic, **α**-helical peptides have recently been recognized as key determinants of CA-MRSA virulence [24]. They are produced by all *S. aureus* strains, except in naturally occurring Agr-defective mutants, in which no PSMs are detectable. HL1, USA300, and USA400 produce comparably high amounts of PSMs, while HA-MRSA often lack pronounced production of PSMs owing to low activity or mutation of the Agr system [13]. Accordingly, all three strains harbor the genetic loci encoding the PSMα peptides PSMα1 through PSMα4, PSMβ1 and PSMβ2, and the δ-toxin, which is encoded within RNAIII of the Agr quorum-sensing virulence regulator. Notably, the *psm*α genes are often not annotated in *S. aureus* genomes, due to their short length, but we annotated them in the HL1 genome and ascertained presence in USA300 and USA400.

Altogether, these findings are in good accordance with the comparable virulence of HL1 and USA400 in the rabbit model of skin infection that we performed previously [13]. The slightly higher virulence of USA300 in that model may be due to ACME-encoded *speG*, which a recent report indicates promotes virulence during skin infection [7]. Furthermore, they are in line with previous epidemiological studies and infection models performed in the PVL-sensitive rabbit indicating that PVL, as the only major virulence determinant that is absent from HL1 and present in USA300 and USA400, does not have a significant impact on virulence during CA-MRSA skin infection [12,13,25].

### SCC*mec*

**Figure 3:**
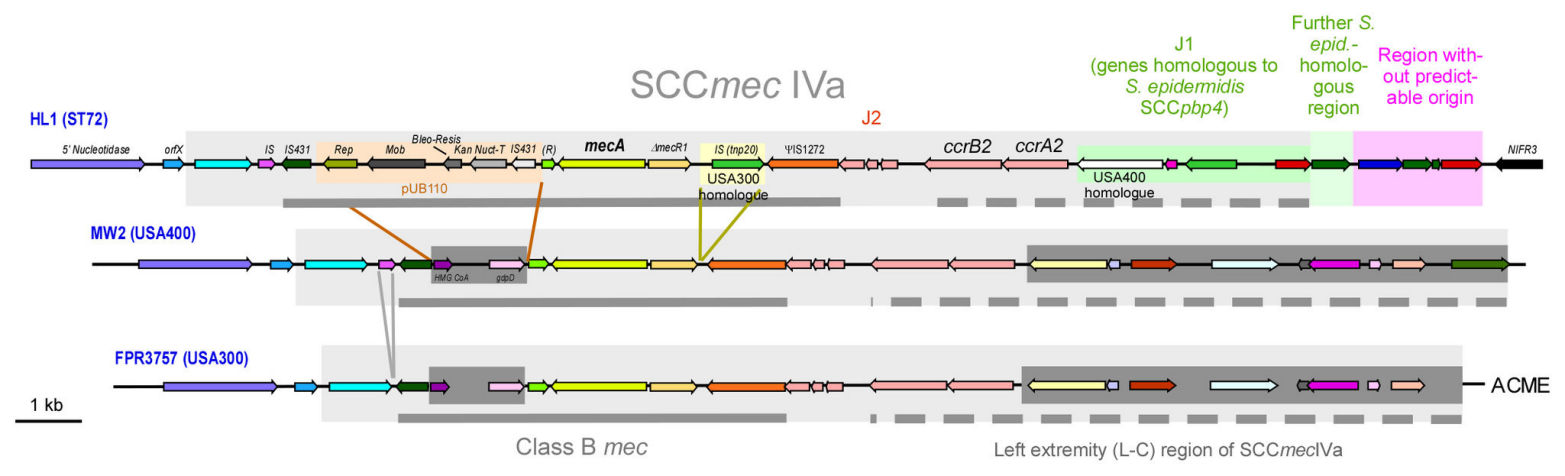
SCC*mec* IVa of ST72 HL1. The HL1 SCC*mec* IVa element and the adjacent unique downstream region are compared to the SCC*mec* IVa elements of USA300 and USA400. SCC*mec* regions are shaded in grey boxes. Colored internal boxes in the case of HL1 show differing regions. Regions present in USA300 and USA400, but absent from HL1 are in dark grey, internal boxes. Solid grey bar, class B *mec* region; dotted grey bar, left extremity (L–C) region. Rep, replication protein; Mob, plasmid mobilization protein; Bleo-Resis, bleomycin resistance protein; Kan Nucl-T, kanamycin nucleotidyltransferase; (R), (R)-specific enoyl-CoA-hydratase; HMGCoA, hydroxymethylglutaryl CoA reductase; ACME, arginine catabolic mobile element (downstream of SCC*mec* in USA300).

The comparison of the HL1 genome with those of USA300 and USA400 only revealed pronounced differences, encompassing a series of genes, in a very limited number of locations. The strongest difference was seen in and surrounding the SCC*mec* IVa element (Fig. 3). As previously described by Park et al., the class B *mec* cassette of ST72 SCC*mec* IVa element contains a *tnp20* IS element and a pUB110 region in addition to the SCC*mec* IVa element of USA300 and USA400 [26]. The *tnp20* IS element is also found at another location in the genome of USA300, but not USA400. The pUB110 region comprises genes involved in kanamycin and bleomycin resistance, in addition to plasmid replication and recombination enzymes. In the left extremity (L-C) region of HL1 SCC*mec*, there are four genes with high similarity to genes found in the *S. epidermidis* ATCC12228 genome, indicating that they may have been acquired from *S. epidermidis* – similar to SCC*mec* IV in general, which is believed to have originated from *S. epidermidis* at an earlier time [27]. One of the genes in this region has a homologue at another location in the USA400 genome, but not in USA300, while the others lack homologues in those strains. The *tnp20* IS element and the USA400 homologue are in vicinity of the *nifR3* gene, encoding a putative nitrogen regulatory protein, in the respective USA300 and USA400 genomes, while the *nifR3* gene is found close to the J1 region of HL1 SCC*mec*, indicating that recombination between these two genomic sites occurred.

### Plasmids

**Figure 4:**
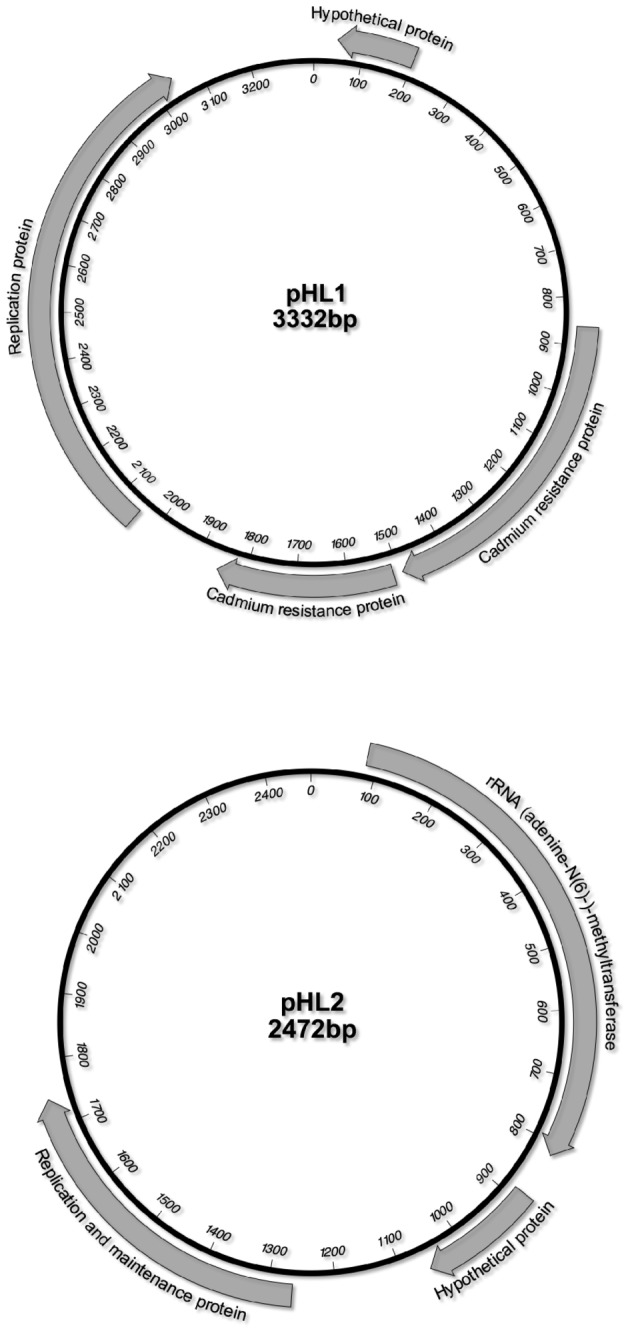
Plasmids in ST72 HL1.

Plasmid pHL1 (3332 bp) contains four ORFs, encoding a plasmid replication protein, a hypothetical protein, and two cadmium resistance proteins (Figure 4). Plasmid pHL2 (2472 bp) encodes a plasmid replication protein, a hypothetical protein, and an rRNA (adenine-N6-) methyltransferase (Figure 4). No virulence factors are encoded on the HL1 plasmids.

### Concluding remarks

Our analysis of a CA-MRSA genome of a previously not analyzed ST advances our understanding of the CA-MRSA pandemic, especially as it represents the first sequenced genome of a PVL-negative CA-MRSA strain. It further strengthens the hypothesis that the success of CA-MRSA as pathogens is multi-factorial rather than dependent on the acquisition of specific, CA-MRSA-characteristic virulence determinants. In particular, we did not find virulence determinants in ST72 with an apparent role of substituting for the absence of PVL, such as, especially, other leukotoxins. While further detailed functional and gene expression analyses will be necessary, these findings suggest that the virulence of ST72 CA-MRSA is mainly dependent, as previously suggested for CA-MRSA in general [5,14], on gene regulatory adaptations enhancing the expression of core genome-encoded virulence determinants.
